# Metasurface Antennas: New Models, Applications and Realizations

**DOI:** 10.1038/s41598-019-46522-z

**Published:** 2019-07-15

**Authors:** Marco Faenzi, Gabriele Minatti, David González-Ovejero, Francesco Caminita, Enrica Martini, Cristian Della Giovampaola, Stefano Maci

**Affiliations:** 1 Department of Information Engineering and Mathematics, University of Siena, Via Roma 56, 53100 Siena, Italy; 2 Wave Up s.r.l., Via Roma 77, 53100 Siena, Italy; 3 Univ Rennes, CNRS, IETR (Institut d’Electronique et de Télécommunications de Rennes) - UMR 6164, F-35000 Rennes, France, 263 Avenue du Général Leclerc, Rennes, 35042 France

**Keywords:** Electrical and electronic engineering, Electronic devices

## Abstract

This paper presents new designs, implementation and experiments of metasurface (MTS) antennas constituted by subwavelength elements printed on a grounded dielectric slab. These antennas exploit the interaction between a cylindrical surface wave (SW) wavefront and an anisotropic impedance boundary condition (BC) to produce an almost arbitrary aperture field. They are extremely thin and excited by a simple in-plane monopole. By tailoring the BC through the shaping of the printed elements, these antennas can be largely customized in terms of beam shape, bandwidth and polarization. In this paper, we describe new designs and their implementation and measurements. It is experimentally shown for the first time that these antennas can have aperture efficiency up to 70%, a bandwidth up to 30%, they can produce two different direction beams of high-gain and similar beams at two different frequencies, showing performances never reached before.

## Introduction

Metasurfaces (MTSs) are thin artificial layers consisting of periodic arrangements of small inclusions in a dielectric host medium. They can be designed to achieve unusual reflection/transmission properties of space waves and/or to modify the dispersion properties of surface/guided waves^1–8^. MTS technology has received considerable attention by the scientific community and has been employed in a number of applications; a recent overview can be found in^9^. In microwave antennas applications, MTSs are usually formed by a regular texture of small elements printed on a grounded slab with or without shorting vias^10–21^. Alternatively, at submillimeter wavelengths, MTSs can be constituted by a dense distribution of metallic pins on a ground plane^22,23^.

When the surface wave (SW) interacts with modulated, locally periodic boundary conditions (BCs) imposed by a MTS, it allows one to reproduce a desired aperture field radiating through a leaky wave (LW) effect^24,25^. The typical MTS structure presents a low profile, weight, and complexity, and can be manufactured with standard printed circuit board techniques. Moreover, the feeding element is embedded in the MTS plane, so that one can avoid external protruding or backing feed arrangements or (sub-) reflectors. These attractive features, combined with the possibility of a fine control and shape tailoring of the aperture field, result in a number of interesting antenna devices recently proposed in literature. Challenging designs have been also made possible through the evolution of MTSs modeling^26^, in the perspective of beam scanning. In^4,27^, Huygens MTSs have been proposed to acquiring additional degrees of freedom in the control of the radiating field. Also, the possibility of obtaining multibeam radiation from a single aperture has been displayed, by coupling a MTS and Luneburg lenses in^28^ or in^29^. Accurate control of the beam polarization has been demonstrated in^16,30^; syntheses of shaped apertures have been proposed in^31,32^. Implementation of MTS structures able to simultaneously support TM and TE radiating modes have been shown in^33^.

The MTSs use elements whose geometries gradually change from cell-to-cell. At the frequency of operation, the elements that implement the impedance BCs are small in terms of the wavelength (typically, between λ/5 and λ/10, with λ being the free-space wavelength). Hence, the interacting SW sees the interface impedance BC as a continuum. The MTS constitutive elements behave like pixels in a black and white printed image, whose gray scale is realized by changing the dimension of the elements inside a regular lattice. The pixels are arranged in a regular lattice with elementary cell size in the range λ/10-λ/5. Usually, the lattice is of Cartesian type, but one could also exploit hexagonal lattices^33^.

**Figure 1:**
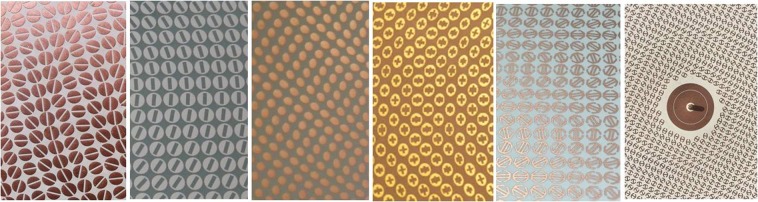
Examples of textured layout for implementing the MTS: (**a**) coffee bean, (**b**) patch with slot, (**c**) grain of rice, (**d**) patch with cross slot (**e**) double π (f) double anchor and details of the feed.

Figure 1(a–f) shows examples of pixels we used to implement the MTS. Despite its discretized nature, the impedance BC formed by the patch texture can be modelled within an excellent approximation as a continuous sheet of anisotropic impedance BC, which supports and modifies the SW propagation.

The SW launcher is simply a coaxially fed vertical monopole and it is located at the center of the circular aperture (see Fig. 1(f)). Usually, the monopole passes through the center of an annular patch loaded with an annular slot. This allows an impedance matching as well as the maximization of the SW power launched on the MTS. The feeding system might also be more complex, e.g., a hat-feed excited by a TM_01_ circular waveguide, to cover a larger bandwidth or to improve the efficiency of SW excitation. However, the SW launched by the feed can be always assumed to be cylindrical and azimuthally symmetric, as the one provided by a monopole. In an efficient feeder, the space field radiated directly by the dipole in free-space should be minimized.

In this paper, we present six new designs and prototypes of MTS antennas which possess high-efficiency (for both broadside and squinted beams), shaped beam, broadband, dual-band and dual-beam, respectively; these prototypes extend considerably the class of applications covered by this technology. Four of these new designs are also implemented in prototypes and the numerical results are validated by measurements with an excellent agreement between theoretical and experimental results.

The paper is organized as follows. The first section describes the electromagnetic models of analysis and synthesis at a different level of complexity. This introduction is important for appreciating the excellent agreement between measurements and models at different levels of complexity. This first section also includes new design considerations on the choice of the elements for the new various applications. The second section presents the new high-performance antenna prototypes and their measurements, and highlights the improvement and novelty with respect to similar solutions present in literature.

## Analytical and Numerical Electromagnetic Models

### Continuous anisotropic reactance model

**Figure 2:**
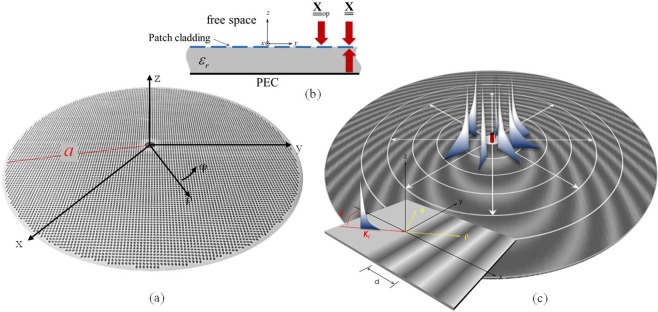
Geometry of the problem: (**a**) the cladding made of subwavelength patches is modelled as a sheet tensor reactance which defines through equation (1) a “transparent” IBC on a grounded dielectric slab (**b**). (**c**) The local periodic problem for the definition of the adiabatic Floquet modes on the continuous reactance sheet.

The type of antennas we will refer to consists of a lossless grounded dielectric slab of relative permittivity ε_r_ and thickness h_d_, printed with perfectly electric conducting (PEC) subwavelength patches within a circular surface of radius a, (Fig. 2(a)). The PEC patches form a penetrable impedance BC (Fig. 2(b)) with modulated capacitive reactance as the one shown in Fig. 2(c). In the description of the geometry, we will use a cylindrical reference system with coordinates (*ρ*, *ϕ*) and unit vectors $(\hat{{\boldsymbol{\rho }}},\hat{{\boldsymbol{\phi }}})$ (from now, bold characters denote vectors, and bold characters underlined by a double bar denote tensors). The origin of this coordinate system is centered at the point where the feeder is located, i.e., at the center of the circular aperture. The metallic cladding constituting the MTS is modelled as a continuous sheet-transition, anisotropic BC^24,34^. Such BC is defined by

1 $${{\bf{E}}}_{t}({\boldsymbol{\rho }})=j\underline{\underline{{\bf{X}}}}({\boldsymbol{\rho }})\cdot \hat{{\bf{z}}}\times ({{{\bf{H}}}_{t}|}_{{0}^{+}}-{{{\bf{H}}}_{t}|}_{{0}^{-}})\doteq j\underline{\underline{{\bf{X}}}}({\boldsymbol{\rho }})\cdot {\bf{J}}({\boldsymbol{\rho }})$$

where $\underline{\underline{{\bf{X}}}}({\boldsymbol{\rho }})$ is the “transparent” homogenized reactance and **J** is the electric current flowing in such a reactance. The transparent *lossless* reactance of interest in our design is referred to as “constant-average reactance”, and its general form is $\underline{\underline{{\bf{X}}}}({\boldsymbol{\rho }})=\underline{\underline{\bar{{\bf{X}}}}}\cdot (\underline{\underline{{\bf{I}}}}+\underline{\underline{{\bf{M}}}}({\boldsymbol{\rho }}))$ where $\underline{\underline{{\bf{I}}}}$ is the identity matrix, $\underline{\underline{\bar{{\bf{X}}}}}$ is a **ρ**-independent diagonal tensor in cylindrical unit vector components $(\hat{{\boldsymbol{\rho }}},\hat{{\boldsymbol{\phi }}})$, and the entries of $\underline{\underline{{\bf{M}}}}({\boldsymbol{\rho }})$ have a zero mean value over the circular surface. These entries have a form of type

2 $${m}_{i,j}({\boldsymbol{\rho }})\cos (K\,s({\boldsymbol{\rho }})+{\Phi }_{i,j}({\boldsymbol{\rho }}))$$

where *i*, *j* denote the indexes of the entries in cylindrical coordinates and *K* is a large ρ-independent constant with dimension [m]^−1^. *K* is such that $K|{\nabla }_{t}s({\boldsymbol{\rho }})|\gg |{\nabla }_{t}{\Phi }_{i,j}({\boldsymbol{\rho }})|$, with the transverse gradient operating on **ρ**; namely, the length *s*(**ρ**) has a slow variation in terms of the wavelength.

### Adiabatic Floquet modes (AFM) model

The Adiabatic Floquet Mode (AFM) expansion is an analytical model to describe currents and fields when an elementary dipole source is placed at the center of the impedance defined in the previous section. The design method based on the AFM model is the key-methodology that allows the antennas presented hereinafter to reach very high-performance. This model, introduced by our group in^24,25^, stems from the rigorous FW expansion of a 1D periodic problem adiabatically extended to the case of locally periodic 2D impedance BCs. Even if the actual impedance BCs are not strictly periodic, the Floquet mode (FM) expansion still provides a good local description of fields and currents associated with the locally periodic surface. Such local periodicity is defined through the gradient of the BC’s phase modulation function, and can be successfully exploited in the MTS design^24,25^.

The AFM expansion for the current is obtained as ${\bf{J}}\approx {\sum }_{n}{{\bf{J}}}^{(n)}$, where the terms of the summation are written as follows^24,25^

3 $${{\bf{J}}}^{(n)}({\boldsymbol{\rho }})={{{\bf{J}}}_{0}}^{(n)}({\boldsymbol{\rho }}){e}^{-jnKs({\boldsymbol{\rho }})}{H}_{1}^{(2)}({\int }_{0}^{\rho }{k}^{(0)}({\boldsymbol{\rho }}{\boldsymbol{^{\prime} }})d\rho {\rm{^{\prime} }})$$

in which ${H}_{1}^{(2)}$ is the Hankel function of second kind and first order, Ks(ρ) is the same as in Eq. (2), and ${k}^{(0)}({\boldsymbol{\rho }})={\beta }_{sw}+{\beta }_{{\rm{\Delta }}}({\boldsymbol{\rho }})-j\alpha ({\boldsymbol{\rho }})$ is a complex wavenumber obtained by a local perturbation of the “unperturbed” wavenumber βsw, the latter obtained from the solution of the dispersion equation formulated only for the radial component of $\underline{\underline{\bar{{\bf{X}}}}}$. In (3) ${{\bf{J}}}_{0}^{(n)}$ are coefficients weakly-dependent on ${\boldsymbol{\rho }}$. Taking the asymptotic form of the Hankel function, it is evident that each mode in Eq. (3) has a curvilinear-wavefront given by ${\int }_{0}^{\rho }{k}^{(0)}({\boldsymbol{\rho }}\text{'})d\rho \text{'}+nKs({\boldsymbol{\rho }})=const.$ The coefficients ${{{\bf{J}}}_{0}}^{(n)}$ in (3) as well as the complex perturbation ${\beta }_{{\rm{\Delta }}}({\boldsymbol{\rho }})-j\alpha ({\boldsymbol{\rho }})$ to the unperturbed wavenumber β_*sw*_ are calculated in a closed form by solving a local problem constituted by a 1D sinusoidally modulated transparent MTS illuminated by an inhomogeneous plane-wave. Therefore, (3) provides a complete closed form asymptotic approximation of the *global* currents.

Among all the Floquet modes of the generalized expansion in (3), only the -1 indexed mode has a significant spectral contribution in the visible region and it is used for a preliminary design of the aperture antenna. The reactance needed to have a certain radiation pattern is obtained by using an alternating projection method by applying iteratively Eq. (3) with 5 modes only (*n* = −2, −1, 0, 1, 2).

### Gaussian–Ring basis functions for MoM analysis by continuous BC (GR-MoM)

A continuous full wave analysis based on continuous BC is used to check the accuracy of AFM. To this end an extremely efficient Method of Moments (MoM) formulation based on Gaussian-Ring (GR) basis functions is used as in^35^. These basis functions are circular rings with Gaussian transverse profile, and azimuthal linear phase variation *exp(-jnϕ)*. Several mathematical tricks allow for having a strongly sparse matrix with entries expressed in a closed form. This allows for a full-wave analysis of 30λ diameter antennas in few minutes on a laptop. Overall, the complete synthesis part is extremely fast (from few minutes to one hour, depending on PC speed and dimension of the antenna).

### Synthesis of the subwavelength printed elements

Once the continuous reactance is synthesized, we proceed to its implementation by means of sub-wavelength patch elements. The element design is carried out by a MoM tool, in which the element features are described with extreme subwavelength basis functions (e.g. λ/50-λ/100). This MoM is formulated in the spectral domain and it implements periodic phase shift conditions. This periodic problem is valid under the “local micro-periodicity” assumption, i.e., by assuming the local element as immersed in a periodic environment of identical elements. The above assumption allows one to apply periodic BCs on the elementary cell boundaries and to use the periodic GF in the integral equation formulation, thus reducing the computational effort to that of a single unit cell. The periodicity of the lattice is always taken as a constant on the aperture, and the variation of impedance is achieved by changing the geometrical parameters. The analysis of the periodic structure, which is inherently extremely fast, is repeated several times in order to construct a database.

Once the database has been constructed, the continuous reactance is discretized using the same unit cell side and shape as in the construction of the database. Next, the database is searched to find the pixel geometry that better implements the impedance value for each surface sample. The outcome is the final pixelated layout of the antenna, where the exact geometry of any single element is defined. The pixelated layout is a discrete implementation of the continuous impedance BCs designed in the previous step.

**Figure 3:**
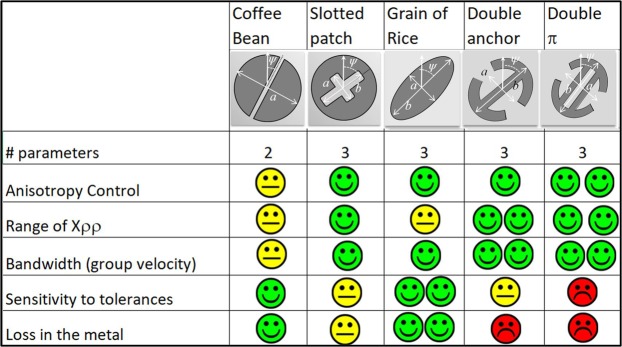
Texture elements features. Summary table with the most relevant electromagnetic performances of the main kinds of textures employed in the synthesis of MTS antennas.

Different geometrical shapes can be used as pixel element. Fig. 3 presents some of them with at least two geometrical parameters, one related to the global dimension (e.g. *a* in the coffee-bean pixel) and one related to the rotation (*ψ*).

The performance of the various elements are presented in terms of anisotropy control, range of variation of X_*ρρ*_, losses in the metal, sensitivity to tolerances and bandwidth. The latter is not related to the geometry itself, but to the capability of the element to load the substrate with a higher capacitance; this allows for printing the elements on a lower permittivity substrate while keeping the dimension of the lattice equal.

Decreasing the substrate permittivity has a benefit in terms of bandwidth, since it implies an increase of the group velocity. The antenna requirements dictate the choice of the element on the basis of this table. The coffee beans (Fig. 3) were suggested in^14^ and^15^. The grain-of-rice and double-anchor elements have been used in the prototypes of the next section. The elliptical shape of the grain-of-rice can be analysed with the quasi-analytical method proposed in^6^, and this enables an extreme speed-up of the data base construction.

### Fast multipole method (FMM) analysis

A detailed analysis of the pixelated layout with a global full-wave solver. The computational effort of simulating the radiation performance of an entire MTS aperture is considerable, due to the large number of pixels (in the order of several tens of thousands) needed to reproduce the impedance pattern. In a MoM framework, this usually translates into a very high number of sub-domain basis functions and, thus, unknowns (in the order of several millions). To overcome the inherent computational challenge, a customized MoM analysis tool has been developed by combining the Fast Multipole Method (FMM) and entire domain basis functions. The Green’s function of the grounded slab is used in the electric field integral equation (EFIE) and a Galerkin testing to convert it in a matrix form $\underline{\underline{Z}}\cdot {\boldsymbol{I}}={\boldsymbol{V}}$. The efficiency of the solver is increased by a FMM approach^36,37^, where the MoM interaction matrix $\underline{\underline{Z}}$ is represented as the superposition of a near interaction matrix ${\underline{\underline{Z}}}_{near}$ and a far interaction matrix ${\underline{\underline{Z}}}_{far}$. The 2D-FMM version^38,39^ is used to accelerate the matrix-vector product relevant to ${\underline{\underline{Z}}}_{far}$, while the near interaction matrix is treated by using a standard MoM technique. More precisely, the ${\underline{\underline{Z}}}_{near}$ entries are calculated by using a spectral MoM, which exploits the efficient procedure in^40^ and the closed-form expression for the basis functions spectra.

The full-wave numerical analysis presented here proves to be agile and accurate: it relies on entire domain functions that considerably reduce the computation time by drastically decreasing the number of unknowns per unit cell^41,42^. As a general rule, one can consider for each element an entire domain basis functions set constituted by only two functions. These functions may have a closed form spectrum obtained by aggregation of transformable basis functions like RWG, and can be able to accurately reproduce the currents on the modulated elements. Furthermore, the closed-form basis functions reported in^6^ can be used for elliptically shaped elements (the grain of rice elements discussed in the previous Subsection). Elliptical patches are versatile elements that feature three geometrical parameters: main axis dimension, axial ratio and on-axis rotation. The available parameters ensure a high anisotropy control, very low sensitivity to tolerances at high frequency and low losses, at the expense of a lower loading capability than other types of elements.

Some comparison examples between this 2D-FMM technique, the “continuous reactance” model and measurements are shown in the next paragraphs dedicated to MTS antennas implementation.

## Realization and Measurements

In this section, we present for the first time examples of antennas designed and realized in projects involving our group, mainly supported by the European Space Agency. These examples are representative of the performance recently achieved with this technology and demonstrate the effectiveness of the design methodology and the accuracy of the analysis methods described in the previous sections.

### Shaped Beam MTS Antenna

**Figure 4:**
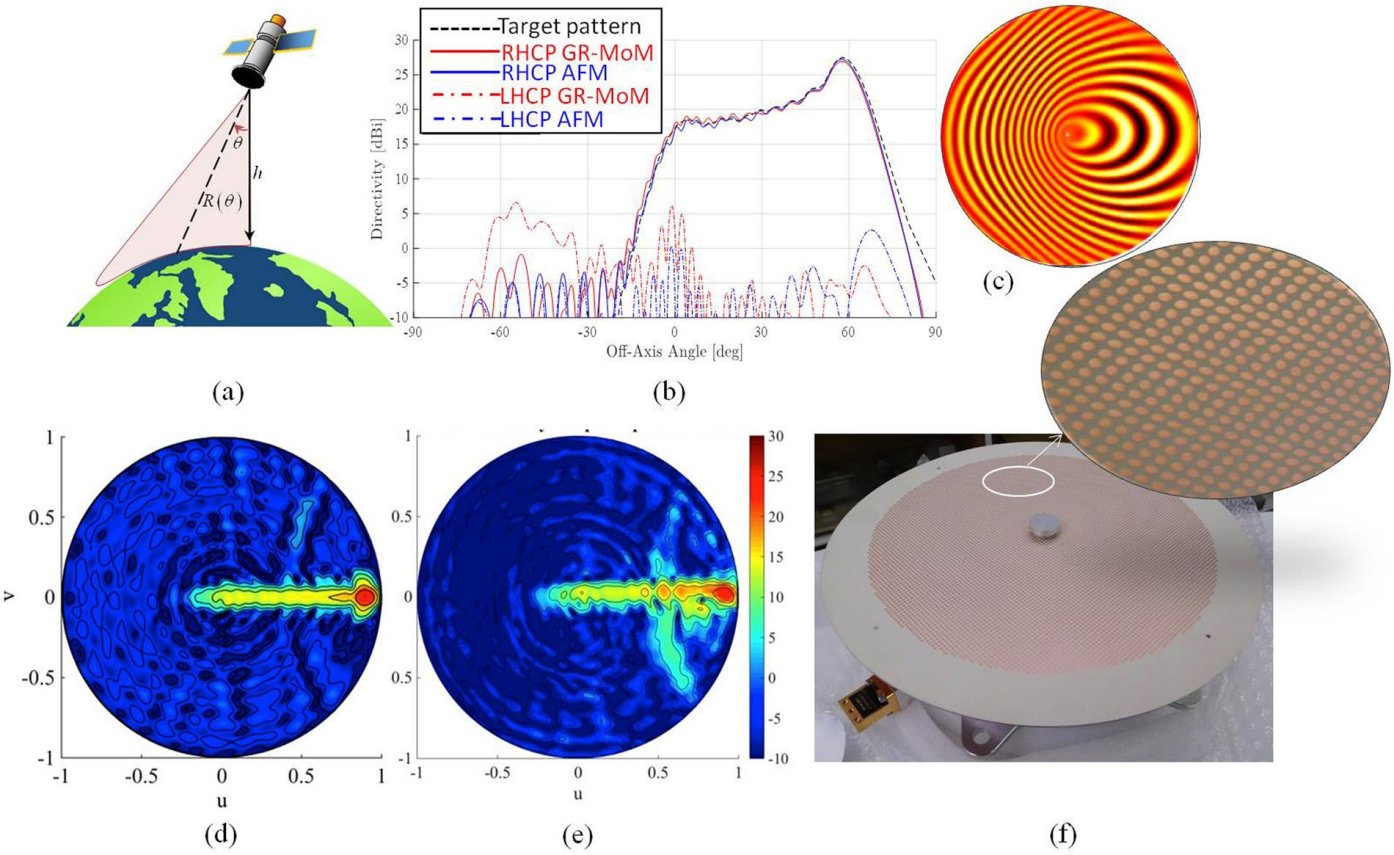
Operative environment, layout and performances of the shaped beam MTS antenna; (**a**) sketch of the Earth surface coverage obtained by using a sectorial isoflux antenna with a turn-table mechanism; (**b**) simulated directivity pattern of a sectorial isoflux MTS antenna for Earth observation missions from LEO satellites; (**c**) sketch of the impedance pattern on the metasurface; (**d**) simulated RHCP component of the directivity for the isoflux sectorial beam antenna in the u-v plane; (**e**) measured RHCP component of directivity for the isoflux sectorial beam antenna in the u-v spectral plane at 26.4 GHz; (**f**) picture of isoflux sectorial beam antenna realization with a detail of the pixelated surface. In the center of the aperture, a metallic cap allows one to improve the antenna matching and the SW launching efficiency.

An isoflux-pattern antenna is presented in Fig. 4. This kind of radiation pattern finds application in data transmission from low-orbit satellites for Earth observation missions. The radiated power distribution with respect to the elevation angle should ensure a uniform power-flux density (isoflux) over the visible Earth surface, as in Fig. 4(a), compensating for differential path loss between Nadir and grazing incidence^14^. When higher gains are required to increase the data rates, a sectorial isoflux profile can be considered: the beam is then squeezed along one spectral direction so that energy is concentrated in a sectorial part of the spectrum to achieve higher gain, while keeping the isoflux profile of the beam (see Fig. 4(b–d)). In this configuration, a mechanical rotation is combined with beam shaping to keep the beam pointing toward the ground station while the satellite moves along its orbit. The concept is sketched in Fig. 4(a). To design the prototype, the target aperture field is determined by an ad-hoc generalization of the procedure presented in^43^ which determines the phase function of an aperture distribution with given amplitude and radiating a desired sectorial beam. Through this method, it is possible to associate to each point on the surface a well-defined direction of radiation. The impedance surface is then synthesized by the AFM method. Figure 4(b) shows the numerically predicted performance of the isoflux pattern antenna whose surface impedance is sketched in Fig. 4(c). Numerical results obtained by the full wave analysis of the continuous impedance surface have been compared with the target beam and with the far field directive pattern obtained by the AFM method. A prototype in Ka-band (26.7 GHz, 2% of relative bandwidth for 2 dB ripple), with right-handed circular polarization (RHCP), has been fabricated on a 0.5 mm thick substrate with 9λ radius at the central frequency and relative permittivity *ε*_*r*_ = 9.8. Numerical and experimental results are presented in Fig. 4(d,e), respectively. The realized prototype is shown in Fig. 4(f): it is fed at the center by a cylindrical waveguide, top loaded with a metallic cap to improve the SW launching efficiency. The feeding structure is realized in aluminum and allows us to rotate the radiative panel.

We remark that for what is relevant to the prototype presented in this section, to the best of our knowledge, this is the first proposed realization of a MTS sector isoflux antenna in the open literature. Isoflux apertures are commonly employed for LEO satellites and can be obtained by choke ring horns^44^. The advantage in implementing a sectorial isoflux is anyway concerned with the possibility of reaching considerably higher gains. Shaped lens antennas have been used to demonstrate sector isoflux patterns also^45^. However, with horns and lens antennas the advantages of obtaining the shaped pattern with high selectivity in azimuth and the extremely simple feeding scheme are compromised.

### Highly Efficient MTS antennas

The fine control of amplitude and phase of the aperture field enables the design of MTS antennas with a high tapering efficiency. The overall efficiency of MTS antennas involves several wave phenomena. In a nutshell, power at the input port is not fully delivered by the feeding system to the SW, since part is directly radiated by the feed in free-space: this introduces a so called feed-efficiency (ε_feed_). Part of the SW power is lost due to losses on metal and dielectric, introducing an ohmic efficiency (ε_Ω_). Additional spurious diffraction at the antenna edges causes a further term of spill-over efficiency (ε_s_). The final gain is related with the radius *a* of the circular aperture as G = (*ka*)^2^(ε_feed_ε_Ω_ε_s_ε_t_) where *k* is the free-space wavenumber and ε_t_ is the conventional tapering efficiency, depending on the amplitude tapering on the surface. This latter parameter is controlled by the modulation index of the surface impedance. A discussion on the relative weights of the various contributions is beyond the scope of this section and can be found in^46^. Here, we present new numerical and experimental results to confirm the limits one can reach.

**Figure 5:**
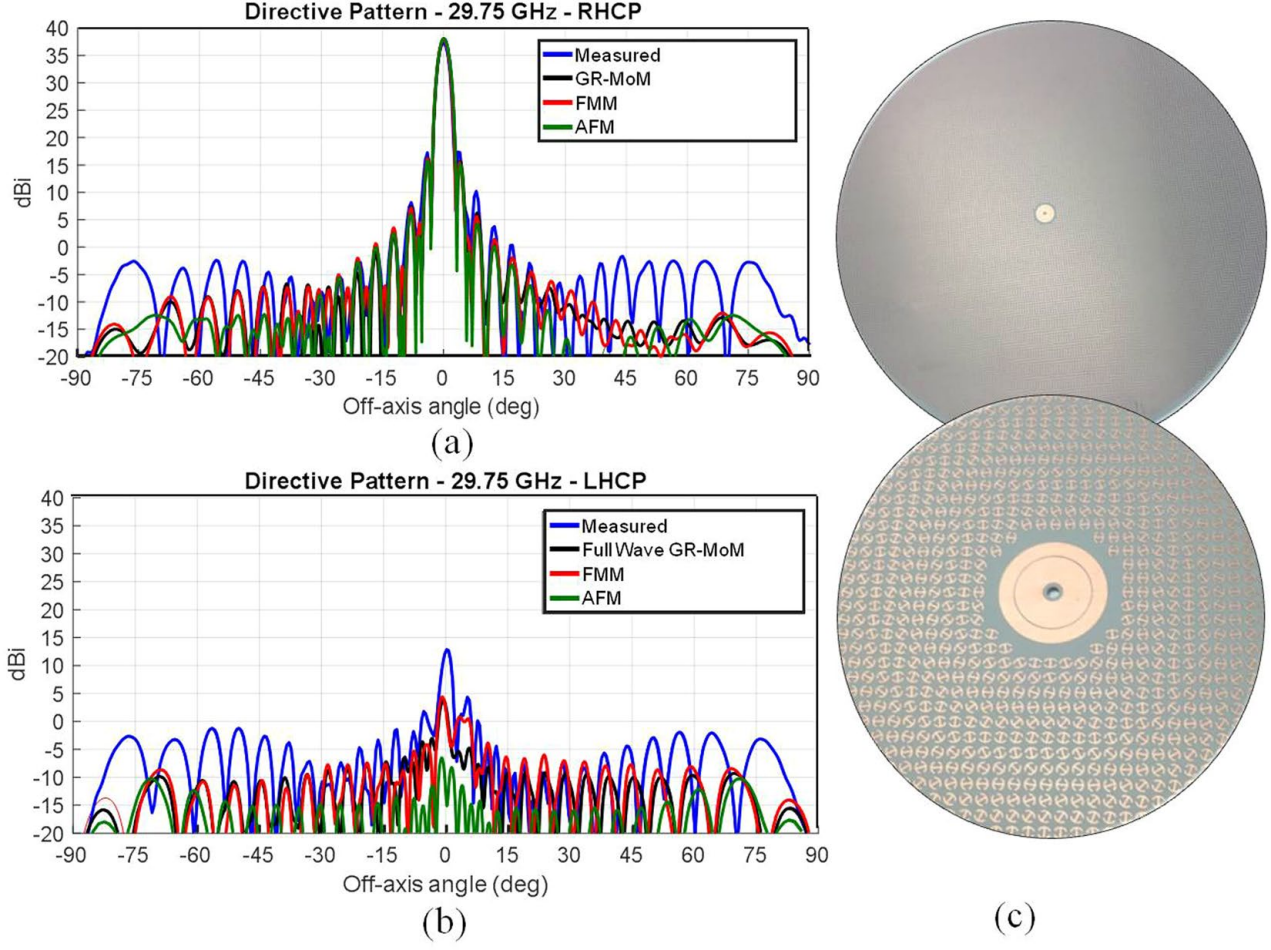
Layout and performances of the high efficiency MTS antenna; (**a**) Co-polar and (**b**) Cross-polar 37 dB directive gain patterns by a MTS antenna at 29.75 GHz designed with product tapering spill-over efficiency of 85%. Curves show a comparison of measurements (Blue line) with three types of numerical predictions. Green-line: AFM method (labelled FO); grey line: GR-basis function continuous impedance BCs MoM^35^; red line: full wave FMM full-wave analysis for the textured layout. (**c**) MTS antenna layout and detail of the feeding region before the insertion of the monopole.

Simulated versus experimental results are presented in Fig. 5(a), which are relevant to an antenna radiating a broadside beam with an aperture of radius 13.5λ at a center frequency of 29.75 GHz. The antenna has been designed with a product between tapering efficiency and spill-over efficiency ε_s_ε_t_ = 85%. The measured gain is 37 dBi, corresponding to an overall efficiency ε_tot_ = ε_feed_ε_Ω_ε_s_ε_t_ of about 70% and a realized product ε_feed_ε_Ω_ of about 80%.

**Figure 6:**
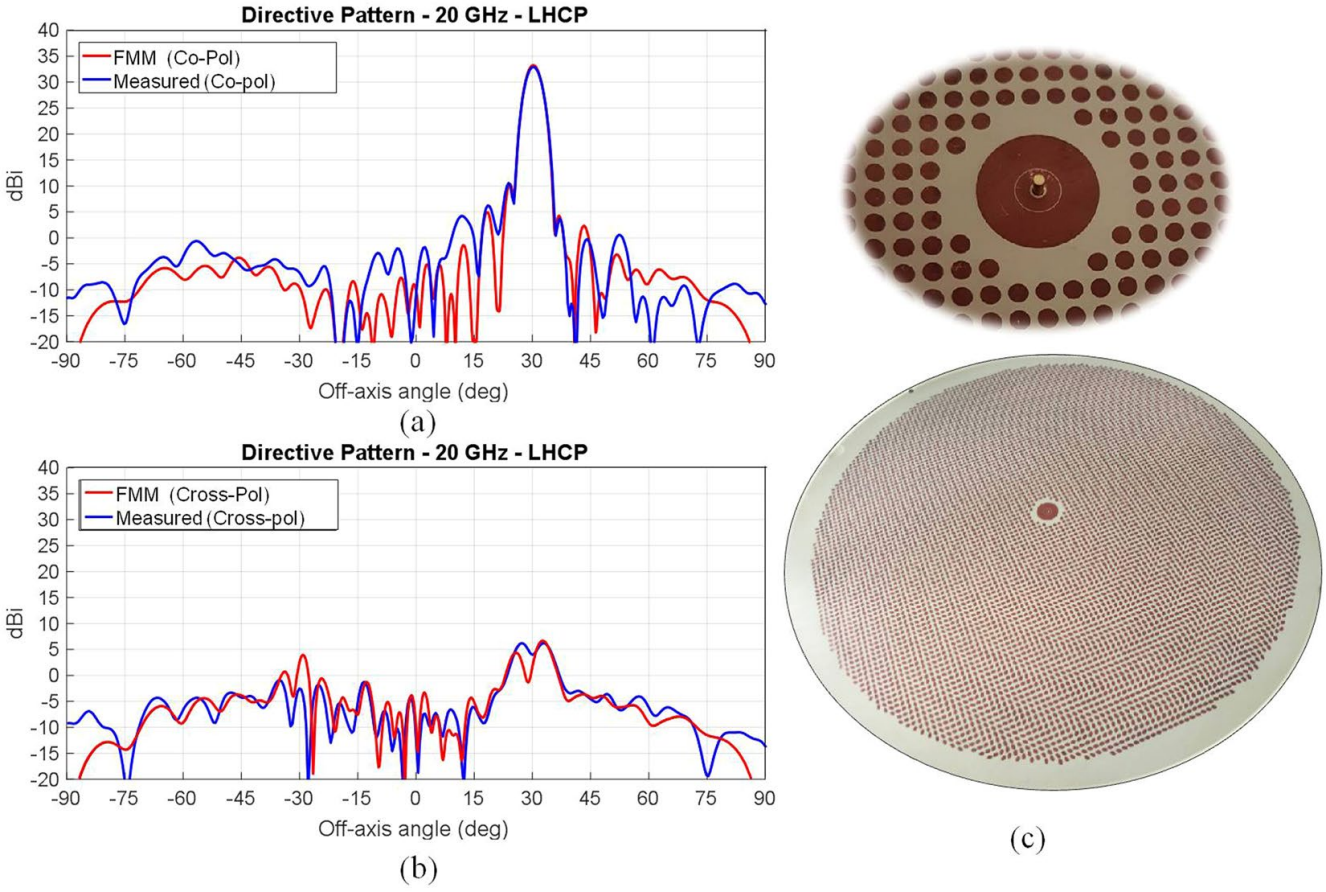
Comparison between measured and simulated directivity of a tilted beam high efficiency MTS antenna realized with printed grain of rice-shaped elements. The antenna has been designed to radiate a RHCP beam tilted 30° from broadside at 20 GHz. (**a**) Co-polar component, (**b**) cross-polar component: comparison between simulated and experimental results; (**c**) Details relevant to the aperture implementation and the feeder structure.

The 3 dB bandwidth goes from 29.1 GHz to 30.5 GHz, corresponding to 4.7% of relative bandwidth, and the axial ratio is better than 1.3 dB within the whole half power beam width. The experimental gain patterns are compared in Fig. 5(a) with three different methods described in the previous section: (*i*) the AFM method (*ii*) the GR-basis function full-wave analysis based on continuous BCs^35^ and (*iii*) FMM full-wave analysis, that accounts for the actual shape of the elements. Figure 5 shows an excellent agreement between the three analysis methods and the experimental results. Figure 6(c) sketches the MTS layouts relevant to the analyzed case; the MTS has been realized on a Rogers RO3003 substrate with thickness 0.762 mm, relative permittivity 3, and dissipation factor tan*δ* = 0.001. This example constitutes a benchmark for the tapering efficiencies that can be obtained by MTS radiators in terms of the directivity.

Figure 6 presents numerical results and measurements relevant to an aperture designed to radiate a RHCP beam tilted 30° from broadside. The antenna has been designed to work at a frequency of 20 GHz and has a radius of 10λ at the center frequency. We can note an excellent agreement between the measured pattern and the one simulated for the textured layout, proving the effectiveness of the design method and the accuracy of the pre-prototyping procedures.

The measured maximum co-polar directivity is 33 dBi, featuring an aperture efficiency of approximately 58%. The 3dB antenna bandwidth goes from 19.65 GHz to 20.3 GHz (about 3.25% of relative bandwidth). In addition, we note a good polarization purity, as the cross-polar component is about 30 dB below the main lobe maximum. The resulting axial ratio is better than 1 dB within the whole half power beam width. The aperture has been realized by modulated grain of rice elements and a detail of the feeding point is also shown in Fig. 6: it is essentially constituted by the inner conductor of a coaxial connector coupled to two concentric annular patches for matching purposes.

### Bandwidth estimate and wideband examples

The bandwidth of MTS antennas modulated with constant period depends on the length of the antenna and on the dispersion characteristic of the surface wave. In^47^ it is seen that the product bandwidth-gain can be estimated for antennas larger than 3 wavelength radius by $GB\approx 22({v}_{g}/c){a}_{\lambda } < 22{a}_{\lambda }$ for uniform amplitude modulation, and $GB\approx 47({v}_{g}/c){a}_{\lambda }/({a}_{\lambda }+2){a}_{\lambda } < 47{a}_{\lambda }$ for optimal amplitude modulation tapering, where G is the gain and $B={\rm{\Delta }}f/{f}_{0}$ is the relative bandwidth, *a*_λ_ is the antenna radius divided by the wavelength at the center frequency *f*_0_, *c* is the speed of light in free space, and *v*_*g*_ is the group velocity of the SW at *f*_0_ for the average impedance. The inequalities in the previous expressions identify a maximum limit of the product bandwidth gain as linearly proportional to the length of the antenna. The bandwidth can be indeed estimated as $B\approx 0.95{v}_{g}/(c{a}_{\lambda }) < 0.95/{a}_{\lambda }$ for a uniform modulation and $B\approx 1,2{v}_{g}/(c{a}_{\lambda }) < 1.2/{a}_{\lambda }$ for non-uniform modulation, namely it is inversely proportional to the antenna dimension.

Both previous expressions are associated to a uniform period of the modulation. Changing the local period is a way to change a larger bandwidth and eventually a wideband operation, at the expenses of a reduction of antenna efficiency. To this end, we observe that any functional design for a wideband broadside beam aperture should be done by matching the local radial periodicity of the frequency-dependent SW wavenumber with the modulation phase *Ks*(*ρ*) in Eq. (2). This can be done by radially shaping *Ks*(**ρ**). The SW wavenumber *β*_*sw*_(*ω*) is related to the impenetrable average reactance ${\bar{X}}_{op}(\omega )$, obtained at a certain frequency as the parallel of the grounded slab contribution and the impedance of the cladding. An optimized design should hence rely on a precise characterization of the dispersion of both the cladding and the slab contributions over the band of interest. Nevertheless, the cladding dispersion is considerably weaker than the one of the slab, and in practical designs the quantity $\underline{\underline{{\bf{X}}}}({\boldsymbol{\rho }},\omega )$ can be just evaluated at one frequency^48^ and then extrapolated to the whole band by a quasi-static extrapolation of the cladding capacitance. If a higher accuracy is needed, one may refine this process as in^49^.

**Figure 7:**
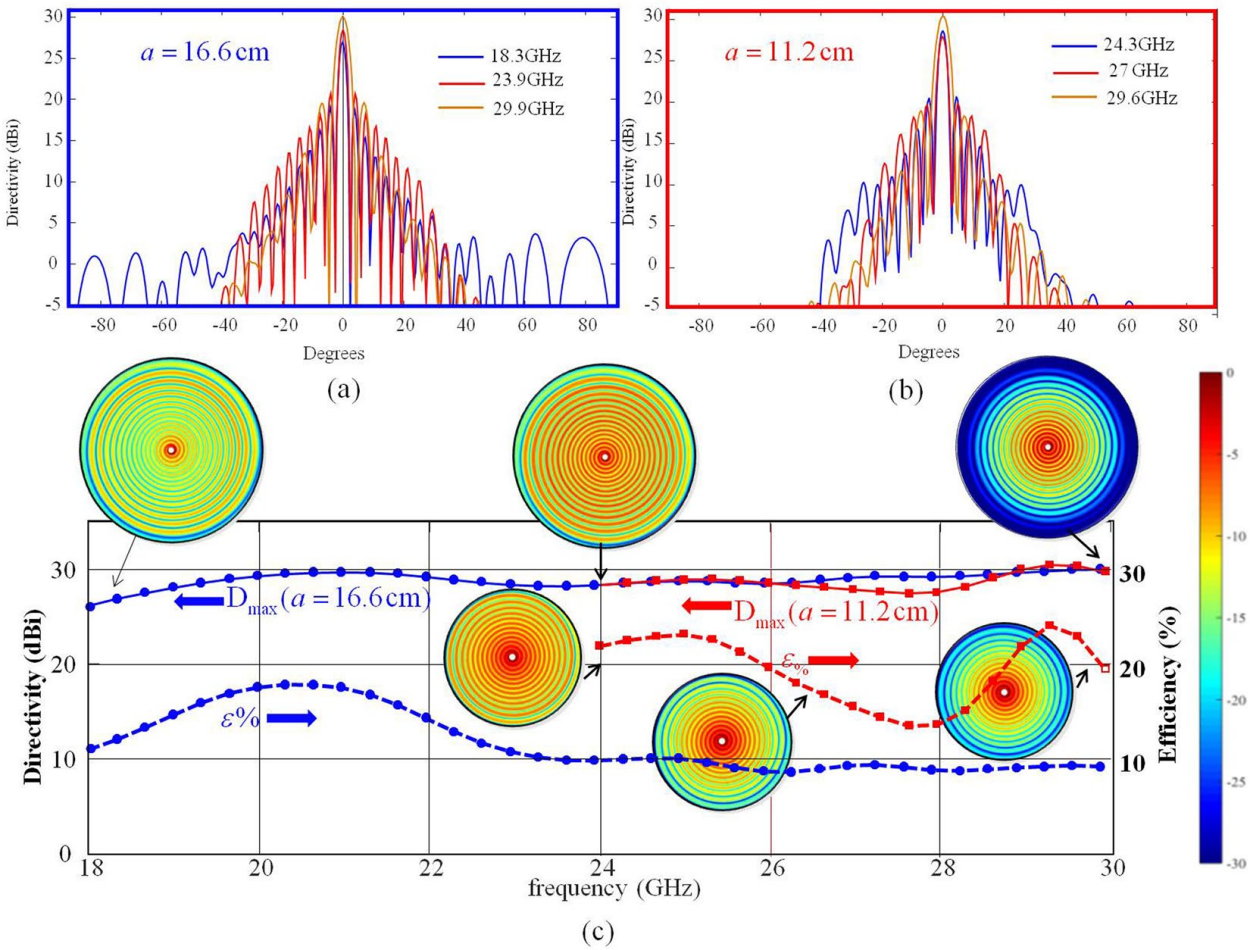
Performances of the wideband MTS prototypes; (**a**) and (**b**) represent directivity patterns obtained with the “continuous impedance“ MoM model for the 16.66 cm radius antenna (**a**) and for a 11.11 cm radius antenna at different frequencies. (**c**) Maximum directivities (solid lines, left scale) and efficiency (dashed lines, left right scale) versus frequency of the two wideband antennas. The insets in (**c**) represent the surface current distribution for both antennas at the same frequency samples used in (**a**,**b**).

The impedance modulation phase *Ks*(*ρ*) can be written as a function of the local period *d*(*ρ*) as $Ks(\rho )=2\pi {\int }_{0}^{\rho }(1/d(\rho ^{\prime} ))d\rho ^{\prime} +Ks(0)$. The function *d*(*ρ*) has an exponential shape which matches at the center and at the antenna contour the SW wavelengths at the limits of the bandwidth; that is ${d}_{1}={d(\rho )|}_{\rho =a}={\lambda }_{sw\max }$, ${d}_{2}={d(\rho )|}_{\rho =0}={\lambda }_{sw\min }$. This way, the aperture is conceived as an active region antenna: by decreasing the frequency, the SW wavelength matches the period in different regions of the surface, so the active region slides outward on the antenna aperture. In the active region, which covers an annular area, the SW is transformed into a LW that radiates a beam in the broadside direction. For the other regions outside the active one, the radiation is very weak, due to the phase mismatch. Figure 7 shows the two different wideband antenna prototypes. In the first example the antenna operates in Ka band in a frequency range comprised between 20 GHz and 30 GHz. In the second example the antenna is instead designed to operate between 24 GHz and 30 GHz. The radii of the two antennas are *a* = 16.66 cm and *a* = 11.11 cm, respectively. Both MTS antennas have been designed on a substrate with relative permittivity ${\varepsilon }_{r}=6.15$ with thickness $h=0.635\,mm$(Rogers RO3006 substrate).

The design frequency of the MTS impedance is fixed at 27 GHz for the first example and 26 GHz for the second one. At these frequencies, the MTSs are characterized by average transparent reactance ${\bar{X}}_{\rho ,\varphi }=-\,450{\rm{\Omega }}$ and ${\bar{X}}_{\rho ,\varphi }=-\,260{\rm{\Omega }}$ (as expressed in Eqs (1,2) and (3–5)). A linear approximation of the admittance variation is considered on the desired bands (from 20 GHz to 30 GHz and from 24 GHz and 30 GHz in first and second examples, respectively) to evaluate the relevant opaque impedances and determine the periodicity functions. A single TM SW launcher, constituted by a vertical electric dipole in the substrate, is placed at the center of the aperture; the antenna in-band responses are calculated using the GR-basis function MoM tool presented in^35^.

Figures 7(a) and (b) show the calculated directivity patterns for both prototypes at three different frequencies. Figure 7(c) shows the frequency response in terms of the calculated broadside directivity and aperture efficiency for both antennas. Both the designed antennas show a flat response in terms of the calculated directivity and good in-band pattern shape stability. It is important to note that the aperture gain will be naturally improved as the requested bandwidth is reduced, as proven in the second example.

In both cases, a broadside pencil beam is maintained within the band of interest, with excellent polarization purity. The inset in Fig. 7(c) shows through the currents distributions how the aperture active region changes over the band. As the operating frequency increases, the illumination is less homogeneous and more confined in the inner region where most of the SW power is radiated away. Conversely, at lower frequencies SW power density reaches the outer region.

The antenna with 16.66 cm radius exhibits a relative bandwidth of 40% (from 20 to 30 GHz) with an average gain of about 29 dB and the antenna with 11.11 cm radius has a bandwidth of 22% (from 24 to 30 GHz) with the same average gain of about 29 dB. These numbers imply a product bandwidth of about 22*a*_λ_ and 17*a*_λ_, for the larger and smaller antenna, respectively, where *a*_λ_ is calculated at the central frequency (25 GHz and 27 GHz, respectively). We stress the fact that we achieved this quite large bandwidth by progressively activating different regions on the aperture, in a similar way as in spirals and log-periodic antennas, but still maintaining the dominant mechanism of surface- to leaky-wave conversion, which ensures a significant gain, that spiral antennas do not have.

### Multibeam MTS antenna

**Figure 8:**
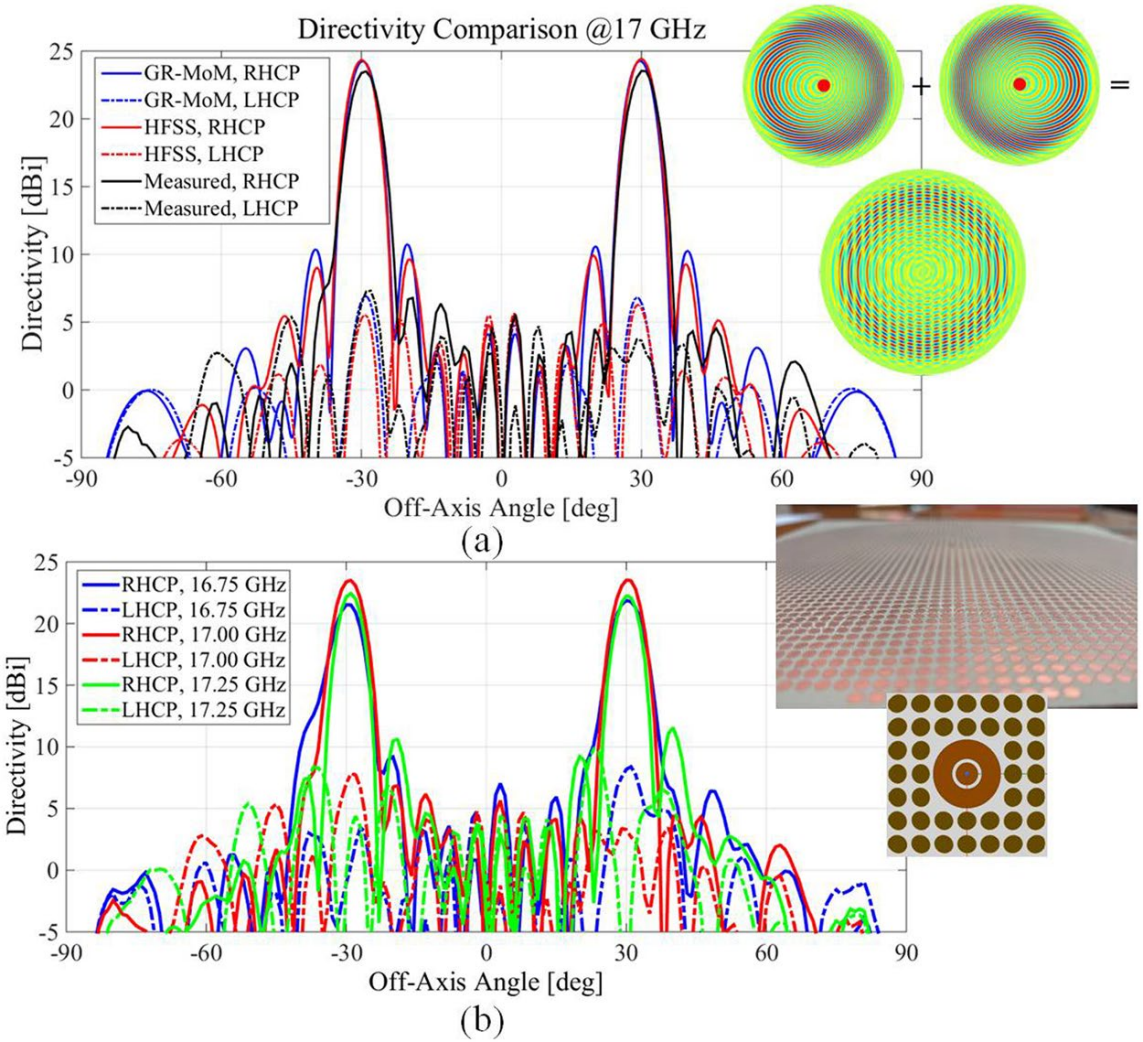
Multibeam MTS antenna radiation performances. (**a**) Simulated (blue line and red line for the IBC-MoM and full-wave results, respectively) and measured (black line) directivity pattern for the multibeam shared aperture MTS antenna at 17 GHz. The obtained beams point in the desired direction, with good cross polar discrimination and beam symmetry. (**b**) Measured directivity patterns at 16.75 GHz, 17 GHz and 17.25 GHz.

Here, the objective is to radiate simultaneous beams with a single aperture in different directions. A simple approach consists in partitioning the radiating aperture and dedicating each partition to one beam. For instance, in^29^ and^30^ multiple beams were generated dividing a circular aperture in several regions. However, the aperture efficiency so obtained is limited by the size of each active area. To overcome this limitation, we propose instead to add the individual modulations that one would require for a single beam at every point of the entire aperture as $\underline{\underline{{\bf{X}}}}=1/2{\sum }_{n=1}^{2}(\hat{{\boldsymbol{\rho }}}\hat{{\boldsymbol{\rho }}}{X}_{\rho \rho }^{(n)}+(\hat{{\boldsymbol{\rho }}}\hat{{\boldsymbol{\phi }}}+\hat{{\boldsymbol{\phi }}}\hat{{\boldsymbol{\rho }}}){X}_{\rho \phi }^{(n)}+\hat{{\boldsymbol{\phi }}}\hat{{\boldsymbol{\phi }}}{X}_{\phi \phi }^{(n)}){U}_{A}$, where ${X}_{\rho \rho }^{(n)},{X}_{\rho \phi }^{(n)}$ and ${X}_{\phi \phi }^{(n)}$ are defined in (Eq. (5)^50^) and U_*A*_ equals one inside the antenna aperture and zero elsewhere. This approach is sketched in the top inset in Fig. 8. Therefore, the whole aperture contributes to the radiation in this approach, and the obtained gains are higher than in an aperture divided in sectors. Moreover, in our prototype, a tapering of the aperture fields is considered in order to increase the aperture efficiency. This approach first presented in^50^ is experimentally validated here.

The antenna has a radius equal to 6*λ* at the center frequency of 17 GHz and it has been designed to radiate two off-axis beams pointing at *θ* = 30° and *θ* = −30°. The aperture is fed by a single source, consisting of a coaxially-fed circular patch, loaded with an annular slot and located at the center of the aperture. The MTS has been implemented by elliptical patches printed on a Rogers RO3010 substrate, which presents a relative dielectric constant ε_*r*_ = 10.2 and thickness *h*_*d*_ = 0.635 mm. Figure 8(a) presents a comparison between the simulated and measured directivity pattern at 17 GHz in the *ϕ* = 0 plane. The simulation of the continuous impedance BC has been carried out with the GR-MoM in^35^, described in the first section, whereas the final structure (consisting of elliptical patches) has been simulated by a commercial full-wave solver (Ansys HFSS). The agreement between measurements and simulated results is very good, as in the previous sections, with two main beams revealed at the expected elevation angles. Each beam has a maximum measured directivity of 23.5 dBi and a 3 dB gain bandwidth of 5.9% for both beams. A good cross-polar discrimination level of −16.5 dBi has been also obtained. The quasi-orthogonality of the beams (and hence their corresponding holographic patterns) enables the proposed approach, validated by this good result. In turn, Fig. 8(b) describes the frequency behavior of this prototype, presenting the directivity patterns measured for the single fed shared-aperture layout at 16.75 GHz, 17 GHz and 17.25 GHz. Only the *ϕ* = 0 cut is presented, although a similar performance has been observed in the other planes. The behavior in terms of the main lobes and beams symmetry obtained for the other two frequency samples is analogous to the one obtained at the central frequency, proving a stable frequency response.

**Figure 9:**
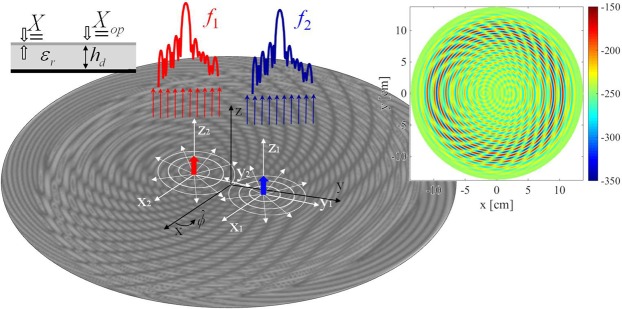
Layout and feeding positioning of the proposed dual band antenna. Position of the feeders are highlighted with the local coordinate systems; each feed is associated to a different frequency. The interference of the two modulation functions is shown in the inset on the upper right inset.

### Dual Band MTS Antenna

**Figure 10:**
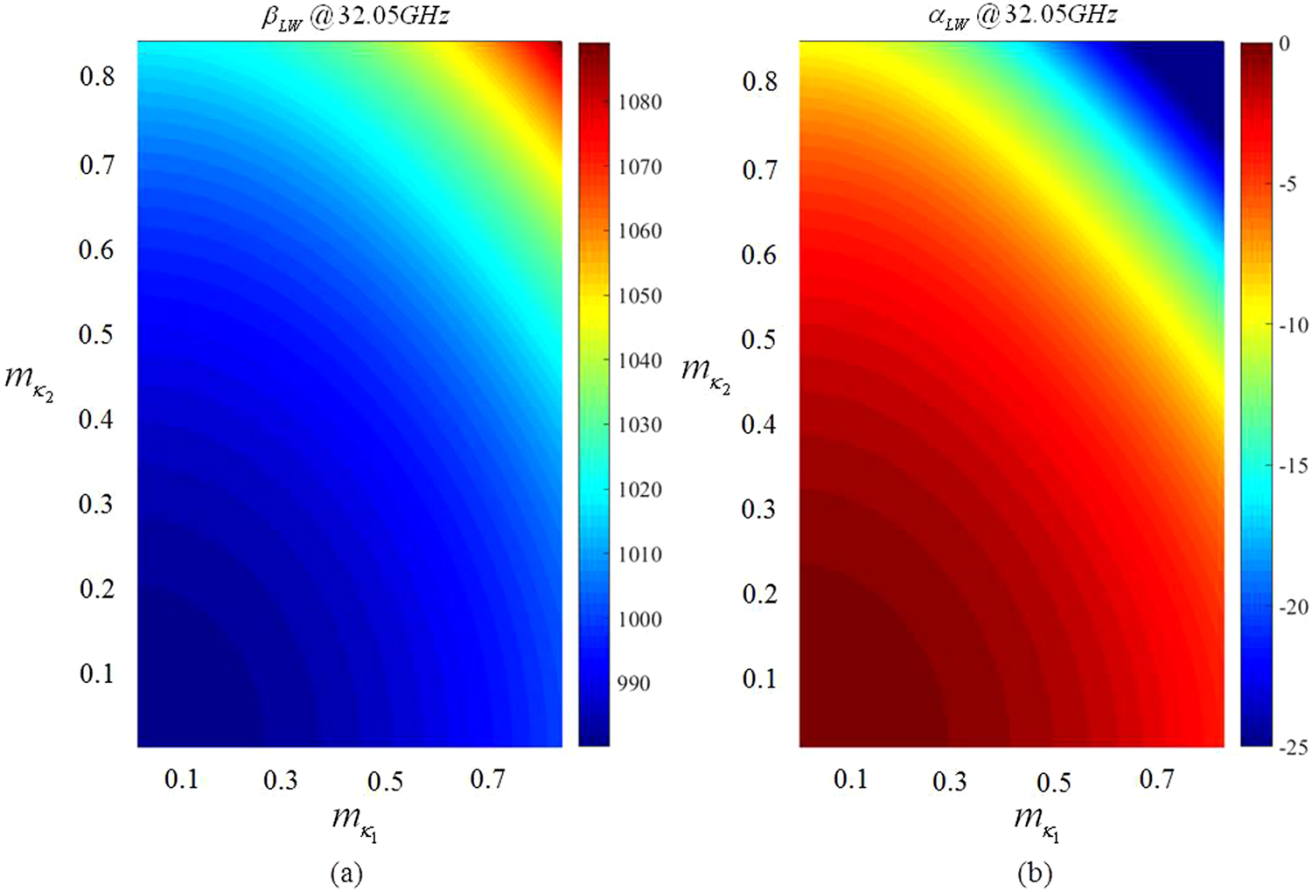
Maps of the leaky wave propagation (**a**) and attenuation (**b**) constants, evaluated at 32.05 GHz. The quantities are expressed as a function of m_κ__1_ and m_κ__2_. The substrate used in this calculations is Rogers RO3010 with ε_r_ = 10.2 and thickness h_d_ = 0.635 mm.

In this Section we introduce a simple design strategy for dual-band modulated MTS antennas. The principle of operation is similar to that described in previous Sections: by periodically modulating an impedance BC, at least one Floquet mode enters in the visible range. To obtain dual-band operation with a single antenna, one needs to tailor a different kind of modulation, and it should be suitable for radiating the SW power in the desired direction at both frequencies. The design strategy presented next is based on the superimposition of two modulations with different periodicity on the antenna aperture. The purpose is to produce beam #1 at frequency *f*_1_ with the first modulation, whereas the second modulation should generate beam #2 at frequency *f*_2_. Once an appropriate surface impedance has been found, analogously as in previous examples, the aperture will be implemented by means of pixels. The design presented here enables also the possibility to decouple the positions of the sources employed to illuminate the antenna at each frequency, so the received signal is self-diplexed. The antenna presented in this section allows one to generate two quasi-orthogonal RHCP broadside pencil beams at K and Ka band. The transparent reactance tensor required to induce the target radiative field reads as (see also Fig. 9)

4 $${X}_{\kappa \kappa }^{(n)}({{\boldsymbol{R}}}_{n})={\bar{X}}_{n}[1+{m}_{\kappa n}({{\boldsymbol{R}}}_{n})\sin ({\beta }_{sw,n}{R}_{n}-{k}_{n}{\hat{{\boldsymbol{r}}}}_{n}\cdot {{\boldsymbol{\kappa }}}_{n}\pm {{\rm{\Phi }}}_{\kappa }^{(n)})]$$

5 $${X}_{\kappa \gamma }^{(n)}({{\boldsymbol{R}}}_{n})={\bar{X}}_{n}{m}_{\gamma n}({{\boldsymbol{R}}}_{n})\sin ({\beta }_{sw,n}{R}_{n}-{k}_{n}{\hat{{\boldsymbol{r}}}}_{n}\cdot {{\boldsymbol{\kappa }}}_{n}\pm {{\rm{\Phi }}}_{\gamma }^{(n)})$$

6 $${X}_{\gamma \gamma }^{(n)}({{\boldsymbol{R}}}_{n})={\bar{X}}_{n}[1+{m}_{\kappa n}({{\boldsymbol{R}}}_{n})\sin ({\beta }_{sw,n}{R}_{n}-{k}_{n}{\hat{{\boldsymbol{r}}}}_{n}\cdot {{\boldsymbol{\kappa }}}_{n}\pm {{\rm{\Phi }}}_{\kappa }^{(n)})]$$

where ${m}_{\chi }({{\boldsymbol{R}}}_{n})=m({{\boldsymbol{R}}}_{n})|{\hat{{\boldsymbol{e}}}}_{n}\cdot {\hat{{\boldsymbol{\chi }}}}_{n}|$, ${{\rm{\Phi }}}_{\chi }^{(n)}={\tan }^{-1}[\text{Im}({\hat{{\boldsymbol{e}}}}_{n}\cdot {\hat{{\boldsymbol{\chi }}}}_{n})/\mathrm{Re}({\hat{{\boldsymbol{e}}}}_{n}\cdot {\hat{{\boldsymbol{\chi }}}}_{n})]$ and $\chi =\kappa ,\gamma$. We notice that the ${\underline{\underline{X}}}^{(n)}$ components are provided in terms of local coordinate systems centered on the feeders. For this reason, the observation point on the aperture is expressed as ${{\boldsymbol{R}}}_{n}={\boldsymbol{\rho }}-{{\boldsymbol{\rho }}}_{n}$, the unit vectors are written as $\hat{{\boldsymbol{\kappa }}}=({\boldsymbol{\rho }}-{{\boldsymbol{\rho }}}_{n})/|{{\boldsymbol{R}}}_{n}|$ and ${\hat{{\boldsymbol{\gamma }}}}_{n}=\hat{{\boldsymbol{z}}}\times \hat{{\boldsymbol{\kappa }}}$. In turn, the quantities ${\bar{X}}_{n}$ and ${\beta }_{sw,n}$ stand for the average transparent reactance and the SW wavenumber at *f*_*n*_, respectively. A space dependent modulation index ${m}_{\chi n}({{\boldsymbol{R}}}_{n})$ has been considered in Eqs (4–6). The modulation index has been synthesized by solving a generalized version of the canonical problem firstly introduced in^51^. The proposed generalization consists in extending the formulation to the case of an anisotropic reactance defined as the sum of two sinusoidal functions with modulation indexes *m*_*χ*1_ and *m*_*χ*2_ (indexes *m*_*γn*_ are posed equal to *m*_*κn*_) and different periods. This new canonical problem is solved for several values of m_χ1_ and m_χ2_, and the corresponding LW wavenumbers determined. Thus, we can establish a mapping between modulation indexes and the LW attenuation constants relevant to composite modulations. Figure 10(a,b) show an example of such mapping for the real and imaginary parts of the wavenumber. The values in Fig. 10(b) are readily available to associate modulation indexes to a suitable field damping profile.

The example at hand (*a* = 9.8 cm, *f*_1_ = 26.25 GHz, and *f*_2_ = 32.05 GHz) has been realized on a Rogers RO3010 with relative permittivity ε_*r*_ = 10.2 and thickness *h*_*d*_ = 0.635 mm. The opaque impedances at frequencies f_1_ and f_2_ are equal to ${X}_{op}({f}_{1})=0.6{\zeta }_{0}$ and ${X}_{op}({f}_{2})=1.1{\zeta }_{0}$, which correspond to transparent reactance values equal to $\bar{X}({f}_{1})=-1058\Omega$ and $\bar{X}({f}_{2})=-796\Omega$. The periodicities of the two functions respect the relation ${d}_{n}=2\pi /{\beta }_{sw,n}$. The aperture has been synthesized by elliptical patches using a best fitting process. The patches are disposed on a periodic Cartesian grid with unit cell side equal to 1.0 mm. The obtained layout has been simulated by a full-wave in-house solver. For this kind of elements^6^ few entire domain basis functions are sufficient to well describe the surface current and they can be integrated into a fast multipole method (FMM) to accelerate the solution (see sections above).

**Figure 11:**
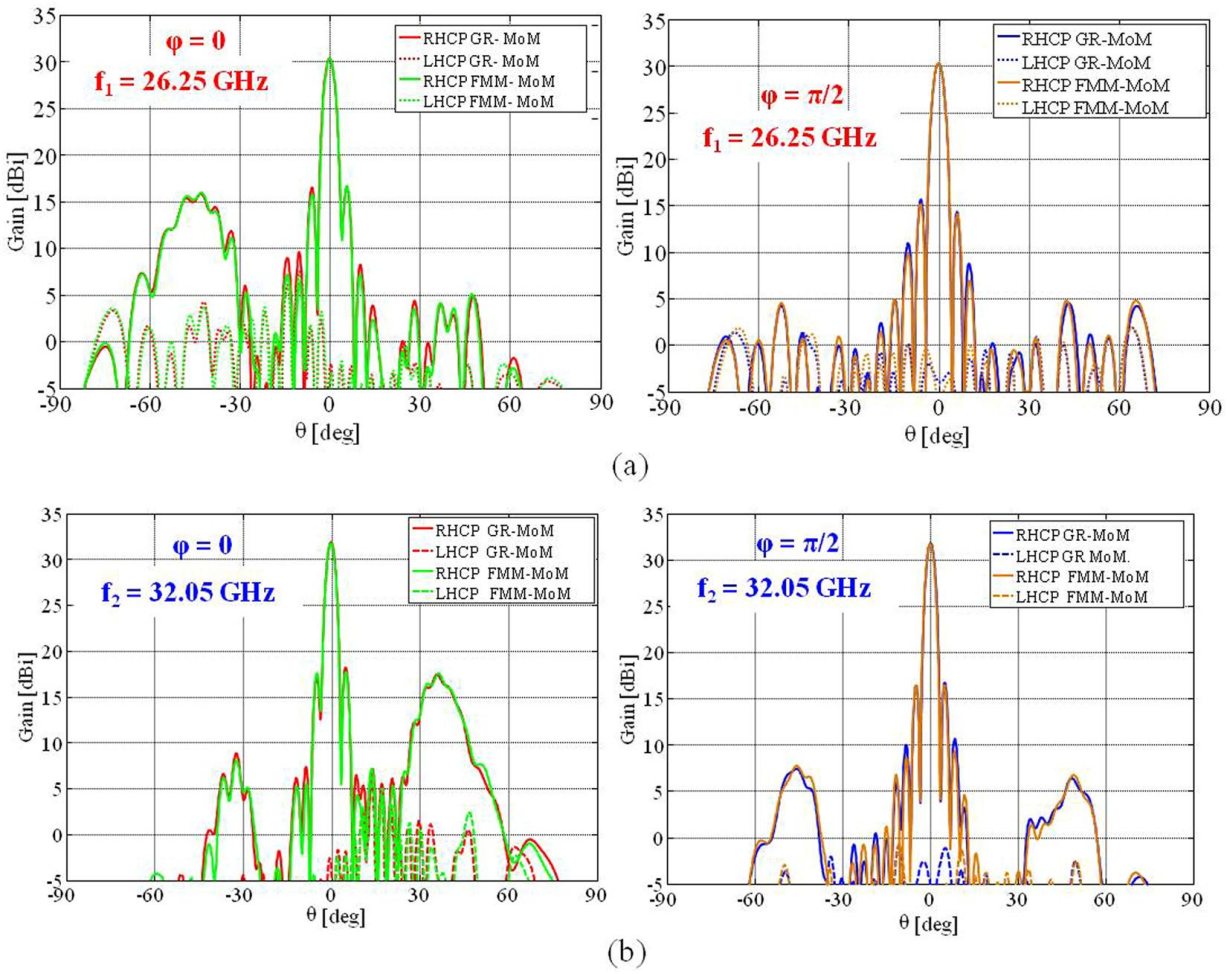
Comparison between the directivity patterns obtained with the GR-basis MoM and the FMM approach that exploits entire domain basis functions for elliptical elements at (**a**) f = 26.25 GHz and (**b**) f = 32.05 GHz. The solid and dashed red and blue lines show the co-polar and cross-polar components, respectively, of the pattern evaluated by the impedance BC-MoM. The solid and dashed green and orange curves present the co-polar and cross-polar components of the radiation pattern, respectively, obtained by the FMM approach.

**Figure 12:**
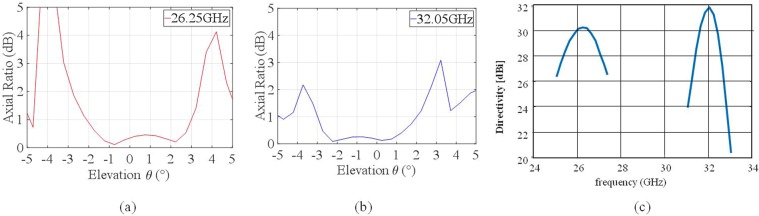
Polarization purity and frequency response of the dual band antenna. Polarization axial ratios near boresight respectively at 26.25 GHz (**a**) and 32.05 GHz (**b**); (**c**) Directivity versus frequency around the two frequencies evaluated by FMM.

Figures 11(a,b) show the obtained directivity patterns at *f*_1_ and *f*_2_. The obtained broadside directivity at *f*_1_ is 30.23 dBi with a very good polarization purity (see also Fig. 12(a) showing the relevant axial ratio), with peak cross polarization levels 23 dBi below the maximum.

A spurious side-lobe appears for *θ* = −40° due to the SW interference with the component of the modulation function introduced to provide a RHCP beam at *f*_2_. At *f*_2_, in turn, the broadside directivity is 31.81 dBi, with peak cross-polarization well below 26 dBi with respect to the Co-Pol maximum (obtained axial ratio is displayed in Fig. 12(b)). The spurious radiation appears in this case for θ = 35°. An excellent agreement is observed between the two analysis methods at both frequencies.

Finally, Fig. 12 presents the axial ratio [(a), (b)] and the frequency response of the structure. The axial ratios of the polarization ellipse in the direction of the main beam, are equal to 0.34 dB at 26.25 GHz and to 0.17 dB at 32.05 GHz, respectively, which is a very interesting level. The 3 dBi directivity percent bandwidth is around ${B}_{ \% }\approx 7.6 \%$ at the lower frequency and around ${B}_{ \% }\approx 3.3 \%$ at the higher frequency, this latter is slightly below the usual performance of single-frequency structures^47^, but extremely significant considering the benefit in the reuse of the same aperture for the two frequencies.

## Conclusions

Metasurface (MTS) antenna design has sprung up and significantly evolved during the last ten years, becoming a significant innovation in the field. In a nutshell, they are a class of leaky wave (LW) antennas in which the energy carried by a surface wave (SW) propagating on an impedance boundary condition (BC) plane is gradually radiated. Indeed, owing to the interaction with the surface impedance, the SW is transformed into a radiative LW whose amplitude and polarization can be finely controlled. The MTS serves to impose the artificially tailored impedance BC seen by the SW, which constitutes the key concept behind MTS antennas. Rather than shaping a conductor, boundary conditions are designed and implemented to obtain the desired radiation characteristics, which include tilted or shaped beams. All this, while preserving the light weight and extremely low profile of the device. Here, we have briefly summarized the design process and we have provided several new examples of MTS antenna designs. We have shown that by properly designing the impedance BC, one can obtain shaped beams or obtain measured aperture efficiencies of 70%. We have also proven that a proper design of the impedance BC allows one to control the antenna bandwidth or to realize an antenna operating on two different frequency bands.
